# The Use of Glycerol-Preserved Corneas in the Developing World

**DOI:** 10.4103/0974-9233.61215

**Published:** 2010

**Authors:** Michael R. Feilmeier, Geoffrey C. Tabin, Lloyd Williams, Matt Oliva

**Affiliations:** 1 Department of Ophthalmology, Division of International Ophthalmology, University of Utah, John A. Moran Eye Center, Salt Lake City, UT 84132, USA; 2 Himalayan Cataract Project, Waterbury, VT 05676, USA

**Keywords:** Corneal Blindness, Corneal Transplant, Deep Anterior Lamellar Keratoplasty, Developing World, Glycerol-Preserved Corneas, International Eye Banking, Keratoplasty

## Abstract

Corneal opacity is the third leading cause of blindness in the developing world and encompasses a wide variety of infectious, inflammatory and degenerative eye diseases. Most caes of corneal blindness are treatable with partial or full-thickness keratoplasty, provided adequate corneal tissue and surgical skill is available. However, access to sightrestoring keratoplasty in developing countries is limited by the lack of developed eye banking networks and a critical shortage of tissue suitable for transplantation. Beyond the developed world, corneal transplantation using fresh corneal tissue (FCT) is further hindered by unreliable storage and transportation facilities, unorganized distribution networks, the cost-prohibitive nature of imported tissue, unreliable compliance with medications and follow-up instructions and inadequate health and education services. Glycerol-preserved corneas overcome many of these limitations inherent to the use of FCT. As surgical innovation in lamellar corneal surgery expands the potential use of acellular corneal tissue, long-term preservation techniques are being revisited as a way to increase availability of corneal tissue to corneal surgeons throughout the developing world. Herein, we discuss the advantages of using and the applications for glycerol-preserved corneal tissue throughout the developing world.

## INTRODUCTION

The World Health Organization defines blindness as visual acuity worse than 3/60.^1^ Using this definition, there were an estimated 45 million people bilaterally blind in 2001 and another 180 million people with bilateral severe visual impairment.^2^ Globally, corneal opacity is the third leading cause of bilateral blindness after cataract and glaucoma, affecting some 7-9 million people, 90% of which live in the developing world.^2^ Furthermore, corneal blindness is often monocular, generating associated disability, which these statistics do not reflect. Corneal causes of blindness encompass a large number of disease processes, including, but not limited to, infectious inflammatory, traumatic and genetic causes. Of these, trachoma is the most common in the developing world.^2^,^3^

**Figure 1:**
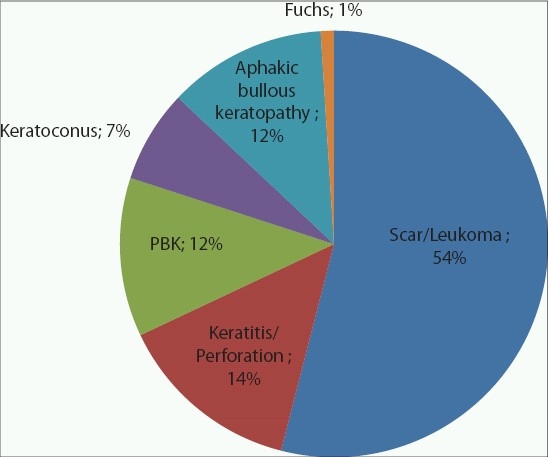
Indication for penetrating keratoplasty in Nepal and India

**Figure 2:**
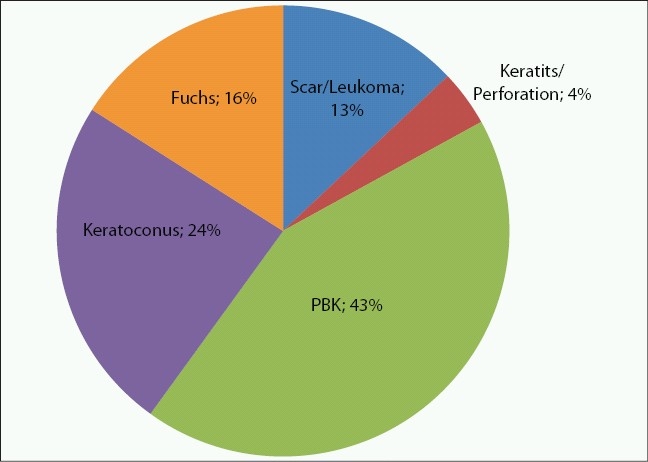
Indications for penetrating keratoplasty in the United States (2001-2005)

Indications for corneal transplantation are markedly different throughout the world, suggesting that geographic and socioeconomic factors greatly influence the etiology of corneal blindness.^2^,^4^ In India and Nepal, corneal scarring and adherent leukoma comprise the over whelming majority of transplant recipients, followed by active infectious keratitis and corneal perforation [Figure 1].^5^,^6^ Significantly less common in these regions are pseudophakic bullous keratopathy, keratoconus and Fuchs’ corneal dystrophy [Figure 1]. In the United States, the three leading indications for penetrating keratoplasty are quite different; pseudophakic bullous keratopathy, keratoconus and Fuchs corneal dystrophy [Figure 2].^7^ Thus, the indications for penetrating keratoplasty (PK) associated with a poor prognosis for graft survival are relatively more common in India and Nepal than in the developed world. In fact, reports indicate that the overall 5-year survival rates for PK are significantly lower in developing countries (49-70%) compared to the developed world (90%)^5^, ^8^,^9^ as a result of differences in surgical indication, unreliable patient follow-up and poor compliance with topical antirejection medications.

Despite these fundamental challenges, keratoplasty is the definitive treatment for corneal blindness and could be utilized to successfully treat an estimated 80-90% of corneal blindness in the developing world. However, successful corneal transplantation requires all of the following: Available high-quality corneal tissue, tissue storage and collection systems, skilled surgeons, antirejection medications and appropriate surgical follow-up. In much of the developing world, access to many or all of these critical components is limited or nonexistent. From a technical standpoint, development of corneal transplantation infrastructure is complex, requiring medical educational systems, technology transfer and infrastructure for eye banking, patient education and cornea collection programs.

In the developing world, increasing the cornea pool to meet the needs of an increasing backlog of patients blinded by corneal opacity and surgeons skilled in transplantation has become a global priority. The importance of self-sustaining local eye banking infrastructure is being realized and significant strategic investments are being made to develop this infrastructure and the human resources necessary to increase the availability of fresh, high-quality cornea tissue.^10^

In addition to these efforts, eye banks throughout the world are evaluating opportunities to maximize the use of the existing corneal pool, and many are revisiting proven long-term corneal preservation techniques, such as glycerol preservation, which are capable of long-term preservation of acellular corneal tissue for up to 5 years.

## BACKGROUND

Kalevar provides a concise and accurate early history of such long-term corneal preservation methods, summarizing the many different lyophilization (freeze-drying) and dehydration techniques reported in the literature.^11^ Many of these methods, although successful in preserving corneal tissue, require relatively expensive equipment and often cumbersome and complex methodology. However, among these early techniques, long-term preservation of corneal tissue using glycerol was simple, yet effective.^12^

Glycerol is a chemical compound commonly called glycerin. Widely used in pharmaceutical formulations, this colorless, odorless and viscous liquid has three hydrophilic hydroxyl groups that impart both its solubility in water and its hygroscopic (water-absorbing) nature. Glycerol is known as a cryoprotectant for storage by freezing and an experimental reagent for corneal permeability studies.^13^ But, as a dehydrating agent, glycerol has antimicrobial and antiprotease properties and maintains corneal structure, making it suitable for long-term storage of corneas for purposes not requiring viable cell layers.^14^–^16^

The pioneering experimental and clinical studies by King *et al*. established the utility of glycerol as an inexpensive and simple means of preserving corneal tissue destined for lamellar keratoplasties.^12^–^15^ The use of glycerol as a means for preserving corneas destined for use in human subjects began in the 1950s with the work of Dr. King, who first used corneas preserved in 95% commercial glycerol under vacuum to ensure an anhydrous state. The sealed dehydrated corneas were stored at room temperature and were used over the following 2 years to provide lamellar grafts to more than 50 patients. The outcomes of the patients in this early study were considered by King to be equal to those obtained using fresh tissue.^14^ King, recognizing the inherent limitations of using a vacuum system, simplified the dehydration process by implementing sodium and calcium aluminosilicates as a desiccant to achieve equivalent levels of dehydration. These molecular sieves act as physical adsorptive agents and remove water to an extremely low vapor pressure, thereby obviating the need for high-vacuum equipment. Corneas preserved using this technique may be stored at room temperature for up to 10 years, after which autolysis of the corneal tissue has been observed.^13^–^15^

Lyophilization was another technique that received significant attention for long-term preservation of acellular corneal tissue. More complex than preservation via glycerol dehydration, lyophilization works by freezing the tissue, followed by reducing the surrounding pressure, then adding enough heat to allow the water frozen in the tissue to sublime. Lyophilization is thought to optimize preservation of corneal architecture. Recent studies using light and transmission electron microscopy found that lyophilization preserved general corneal structure, parallelism of the collagen fibers, Bowman's layer and the epithelial basement membrane.^17^,^18^ No comparative studies have yet determined if lyophilized and glycerol-dehydrated tissues exhibit ultrastructural differences following tissue rehydration and, if detected, to what extent these differences affect tissue quality and corneal transparency. Such information would help determine if lyophilization or other cryopreservation techniques offer advantages that justify the extra cost and equipment.

## ADVANTAGES IN THE DEVELOPING WORLD

Corneal transplantation has several limitations in the developing world. Possibly the single most prohibitive factor is limited access to high-quality cornea tissue. In turn, limited tissue availability is due to lack of eye banking infrastructure, unreliable storage and transportation facilities, limitations in tissue shelf-life, unorganized distribution networks and the prohibitive cost and customs difficulties associated with imported tissues. In addition, access to conventional storage media such as Optisol GS, Eusol C^TM^ or MK media may be impossible or prohibitively expensive. In some regions with access to fresh corneal tissue (FCT), the lack of modern operating rooms and a capable surgical staff, along with poor follow-up and patient compliance with long-term immunosuppressive medications, may limit the long-term success of corneal transplantation. Glycerol preservation addresses many of these limitations, making it highly suitable for certain indications not requiring viable endothelium in the developing world.

First, the use of glycerol-preserved corneas increases the pool of corneas acceptable for transplantation. In 2008, the Eye Bank Association of America (EBAA, www.restoresight.org) estimated that just over 92,000 corneas were harvested in the United States. Approximately 33% of these corneas from medically eligible donors were deemed unsuitable for optical grafting due to an inherent defect. Of these 30,000 unsuitable corneas, approximately one-quarter are suitable for preservation with glycerol and eventual use, given the proper indication. In the United States alone, the use of glycerol as a means of preservation could increase the cornea donor pool by 7,000-8,000 tissues annually.

Glycerol preservation significantly lowers the cost of corneal tissue. In regions where corneal surgical expertise is available, but FCT is not, the high cost (up to $2,000 USD) of importing corneal tissue is often prohibitive. Fortunately, as a result of non-profit entities, such as Global Sight Network™ (GSN) (www.globalsightnetwork.org) (a service of the Alabama Eye Bank), glycerol-preserved corneal tissue is increasingly available for a fraction of the cost of FCT.

Glycerol-preserved tissue overcomes the logistical and technical challenges of tissue delivery and storage. In developing countries with poor infrastructure and unreliable distribution networks, delivery of corneal tissue to the desired destination may take several days or weeks. With a shelf-life of approximately 14-28 days, delivery of FCT presents a significant logistical challenge in the developing world. In addition to a limited shelf-life, proper refrigeration of corneal tissue *en route* and upon arrival is not always possible in areas of the developing world. Glycerol-preserved corneas have the advantage of a 5-year shelf-life (the expiration date of the glycerol) and do not require refrigeration. Not only does this overcome the challenges of safe delivery FCT, it also allows for corneal surgeons throughout the developing world to carry an inventory of acellular corneal tissue suitable for certain indications, including lamellar transplantation and tectonic or therapeutic keratoplasty.

Transplantation using glycerol-preserved corneas may be associated with lower risk of transplant rejection.^19^,^20^ Graft rejection due to poor patient follow-up and non-adherence to immunosuppressive medication regimens is problematic in the developing world following corneal transplantation.^5^ The cellular components inherent to FCT, including epithelium and keratocytes, are sources of major histocompatibility complex antigens that can lead to activation of an indirect immune transplant rejection pathway. Furthermore, in contrast to FCT, glycerol-preserved tissue lacks antigen presenting cells and, therefore, cannot directly sensitize the recipient *t*-cells. As such, acellular corneal tissue, including glycerol-preserved or lyophilized corneal tissue, may significantly reduce the incidence of graft rejection after lamellar keratoplasty, obviating the need for long-term topical immunosuppressive agents and improving long-term transplant success.^19^,^20^

## APPLICATIONS IN THE DEVELOPING WORLD

**Figure 3:**
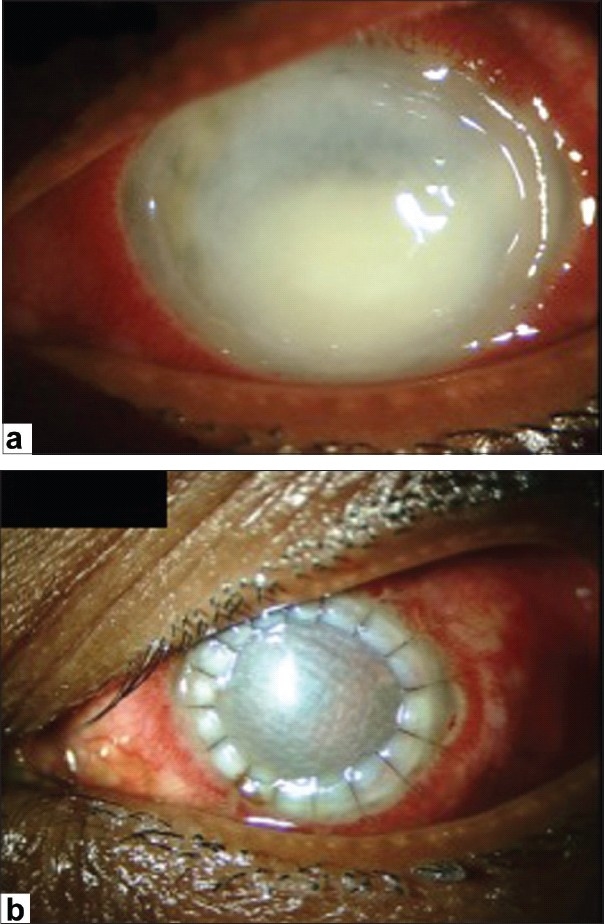
Pre-and post-operative day 1 pictures of a patient with advanced fungal keratitis treated with full-thickness therapeutic keratoplasty using glycerol-preserved tissue

**Figure 4:**
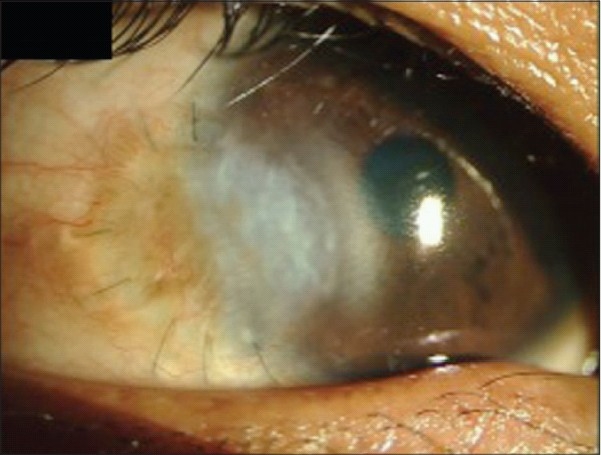
Glycerol-preserved cornea used as a patch graft to treat a patient with a limbal dermoid

**Figure 5:**
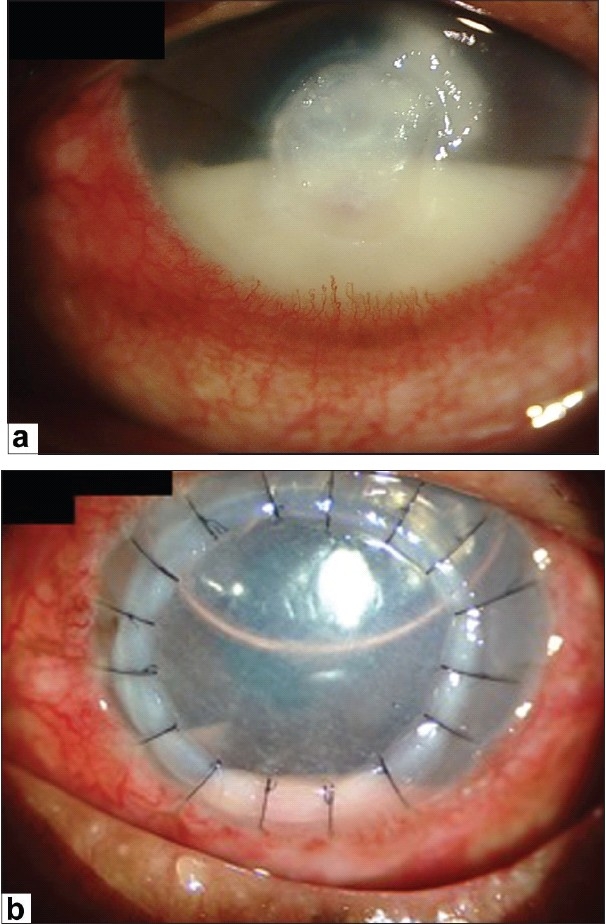
Pre-and post-operative day 1 pictures of a patient with advanced fungal keratitis treated with therapeutic deep anterior lamellar keratoplasty using glycerol-preserved tissue

Although initially successful, the use of glycerol as a corneal preservative was largely supplanted by the introduction and availability of intermediate-term storage media such as Optisol GS and Eusol-C for tissues destined for optical keratoplasties. These storage media have the clear advantage of supporting viable cells, including endothelium, epithelium and keratocytes. Nevertheless, glycerol-preserved tissue is experiencing resurgence in the literature and the clinical indications for its use continue to expand with the increasing popularity of lamellar corneal surgery. Recently, glycerol-preserved corneas have been successfully used for therapeutic and tectonic penetrating grafts [Figure 3], clear small diameter eccentric penetrating keratoplasty, patch grafts [Figure 4], glaucoma tube shunt surgery and for deep anterior lamellar keratoplasty (DALK) [Figure 5].^20^–^25^

Indications for corneal transplant in the developing world differ significantly from those in the industrialized nations. In a retrospective study by Tabin *et al*., of 473 consecutive penetrating keratoplasty surgeries at the Tilganga Eye Institute in Kathmandu, Nepal, the most common indications were corneal scar (37%), adherent leukoma (35%), perforation or impending perforation (9%), pseudophakic bullous keratopathy (6%) and keratoconus (4%) [Figure 1].^5^

As demonstrated by Tabin *et al*., the majority of corneal blindness in the developing world is due to anterior corneal opacification or keratoconus, conditions in which the host cornea typically maintains a healthy endothelial cell layer. DALK, using either a pneumatic dissection (big-bubble technique) or deep manual dissection, is rapidly gaining acceptance as the procedure of choice for such patients, thereby preserving the patient's endothelium.

Other advantages of DALK over PK include improved graft longevity, significantly less graft rejection and improved wound stability. DALK using FCT has been demonstrated to successfully treat corneal dystrophies, corneal scars, keratoconus and acute non-perforated infectious keratitis.^26^,^27^

DALK does not require donor endothelial cells and, therefore, it is the leading application for the use of acellular glycerol-preserved tissue. Chen *et al*. have recently demonstrated the success of using glycerol-cryopreserved tissue in DALK procedures for treating anterior corneal opacity and advanced keratoconus.^20^ In this retrospective consecutive case-series analysis of 48 patients who underwent DALK for stromal opacity, 22 patients under went DALK with FCT and 26 patients with glycerol-cryopreserved corneal tissue (GCCT). This study evaluated the best-corrected visual acuity (BCVA), slit-lamp appearance, corneal topography, pachymetry and laser scanning *in vivo* confocal microscopy at 2 weeks and 1, 3, 6, 12, and 24 months post-operatively.

The study demonstrated no significant differences in spherical equivalent (*P = *0.37), astigmatism (*P = *0.26), BCVA (*P = *0.64), central corneal thickness (*P = *0.73) or endothelial density between the two groups at 24 months. Furthermore, no significant difference in the regeneration of nerve fibers was found in the sub-basal layer and anterior stroma between the two groups at 24 months. Although the keratocyte density was non-detectable at 2 weeks post-operatively in the GCCT group, the density increased significantly over the 24 months postoperatively, but never reached the keratocyte density measured in the FCT recipients.

In this study, one patient in the FCT group demonstrated early graft rejection, while zero patients in the GCCT group experienced rejection. Although epithelial and stromal rejection in DALK is not as frequent as endothelial rejection associated with full-thickness keratoplasty using FCT, it is not uncommon. As previously discussed, the acellular nature of glycerol-preserved tissue decreases the possibility of immune rejection in the setting of DALK. In the developing world, where access to routine follow-up and antirejection medications are limited, this potential advantage must not be overlooked. Although there is science to support this notion,^19^ large comparative studies are needed to determine the extent to which acellular tissue reduces the incidence of stromal and epithelial rejection and whether antirejection medications are necessary in the early and late post-operative period in such patients.

Glycerol-preserved tissue has important indications beyond lamellar surgery, most notably in emergency settings where surgeons are without reliable access to FCT. Emergent keratoplasty is quite common in the developing world. In fact, 10% of all corneal transplants in Nepal are performed on an emergent basis for treatment of severe infectious keratitis or for an acute corneal perforation or an impending perforation. This group of patients represents an ideal setting in which to utilize the glycerol-preserved corneas. Having access to corneal tissue in these emergency settings, corneal surgeons in the developing world can reliably provide eye-salvaging and stabilizing procedures for patients who might otherwise lose their sight and possibly their eye. Furthermore, patients receiving emergency therapeutic transplantation with FCT for severe infectious keratitis have a poor prognosis and a significantly decreased 5-year transplant survival rate. While a full-thickness transplant using glycerol-preserved tissue in such patients is unlikely to yield visual acuity better than hand motion, it does provide a means of globe stabilization until the infection is eradicated and the inflammation is resolved. Following stabilization of the eye, repeat keratoplasty can be performed on an elective basis for optical purposes. In the setting of controlled infection and inflammation, the patient experiences a greater likelihood of success, allowing the most thoughtful and successful utilization of a precious resource, locally obtained, high-quality FCT.

Uses for glycerol-preserved corneal tissue have been demonstrated outside the scope of corneal surgery. Recent reports indicated usefulness for glycerol tissue as patch grafts in primary glaucoma tube shunt surgery and for repair of exposed glaucoma drainage implants.^21^,^28^ In this setting, the corneal transparency associated with glycerol-preserved tissue allows improved visualization of the tube ligature, facilitates laser suture lysis and provides a cosmetically pleasing appearance, offering significant advantages over other non-transparent biomaterials.

## CURRENT AVAILABILITY

Non-lyophilized, glycerol-preserved corneas are currently available through GSN, a non-profit organization founded in June 2008 by the Alabama Eye Bank as a national collection center for corneas from medically eligible donors that are deemed unsuitable for optical keratoplasty. The collaborating eye bank collects the corneal tissue according to their protocol and sends the tissue to GSN. Tissues acceptable to GSN are free from infiltrates and radial keratotomy scarring and meet EBAA donor eligibility requirements. Corneas with any size clear zone, any cell count, other refractive surgery and pterygium are accepted and further screened. Since formal launching at the EBAA fall meeting in November 2008, GSN has received over 2,600 tissues (whole and half corneas) from 18 participating eye banks. Once tissues are received, suitability is confirmed for one of three procedures (anterior lamellar keratoplasty, glaucoma shunt, tectonic graft), and the cornea is placed (whole or divided) into sterilized vials containing anhydrous glycerol and molecular sieves for a quarantine period of 10 days. These corneas are then distributed to corneal surgeons throughout the developing world.

## CONCLUSION

Constraints of availability, cost, storage and transportation may be substantially alleviated by the improved availability of glycerol-preserved corneas in the developing world. Although the list of indications for the use of acellular glycerol-preserved corneas continues to expand, already, they have been proven useful in a variety of therapeutic and optical transplant procedures with levels of success similar to those achieved with FCT.
